# Drake’s Discourses

**Published:** 1852-03

**Authors:** 


ARTICLE IX.

*Dr. Drake's Discourses before the Cincinnati Medical Library Association, Jan. 9th and 10th, 1852.*

We are under obligations to the distinguished author for a copy of this neat duodecimo volume of ninety-three pages.

We commenced reading it as soon as the envelope was torn off and scarcely laid it down until through; though obliged to carry it with us to visit two restless patients who would not be put off. The subject of the first discourse is the history of the early Physicians, Scenery and Society of Cincinnati.

Amongst the first were some noble specimens. The author himself, the only sample left for our inspection, would forcibly impress us with this idea.

The discourse is full of interest and rescues from oblivion a piece of valuable history which no other author could have written.

The second discourse is on Medical Periodical Literature and Medical Libraries. Like the first, it is full of interesting facts and sound reflections.

